# Both Positive and Negative Selection Pressures Contribute to the Polymorphism Pattern of the Duplicated Human *CYP21A2* Gene

**DOI:** 10.1371/journal.pone.0081977

**Published:** 2013-11-29

**Authors:** Julianna Anna Szabó, Ágnes Szilágyi, Zoltán Doleschall, Attila Patócs, Henriette Farkas, Zoltán Prohászka, Kárioly Rácz, George Füst, Márton Doleschall

**Affiliations:** 1 3rd Department of Internal Medicine, Semmelweis University, Budapest, Hungary; 2 Department of Pathogenetics, National Institute of Oncology, Budapest, Hungary; 3 Molecular Medicine Research Group, Hungarian Academy of Sciences and Semmelweis University, Budapest, Hungary; 4 “Lendület” Hereditary Endocrine Tumours Research Group, Hungarian Academy of Sciences and Semmelweis University, Budapest, Hungary; 5 2nd Department of Internal Medicine, Semmelweis University, Budapest, Hungary; University of Lausanne, Switzerland

## Abstract

The human steroid 21-hydroxylase gene (*CYP21A2*) participates in cortisol and aldosterone biosynthesis, and resides together with its paralogous (duplicated) pseudogene in a multiallelic copy number variation (CNV), called RCCX CNV. Concerted evolution caused by non-allelic gene conversion has been described in great ape *CYP21* genes, and the same conversion activity is responsible for a serious genetic disorder of *CYP21A2*, congenital adrenal hyperplasia (CAH). In the current study, 33 *CYP21A2* haplotype variants encoding 6 protein variants were determined from a European population. *CYP21A2* was shown to be one of the most diverse human genes (HHe=0.949), but the diversity of intron 2 was greater still. Contrary to previous findings, the evolution of intron 2 did not follow concerted evolution, although the remaining part of the gene did. Fixed sites (different fixed alleles of sites in human *CYP21* paralogues) significantly accumulated in intron 2, indicating that the excess of fixed sites was connected to the lack of effective non-allelic conversion and concerted evolution. Furthermore, positive selection was presumably focused on intron 2, and possibly associated with the previous genetic features. However, the positive selection detected by several neutrality tests was discerned along the whole gene. In addition, the clear signature of negative selection was observed in the coding sequence. The maintenance of the CYP21 enzyme function is critical, and could lead to negative selection, whereas the presumed gene regulation altering steroid hormone levels via intron 2 might help fast adaptation, which broadly characterizes the genes of human CNVs responding to the environment.

## Introduction

Duplications of individual genes together with their chromosomal regions have long been considered as the primary source to yield novel gene functions [1]. The models concerning the emergence, maintenance and evolution of duplicated genes rely on positive selection to impart new functions [2] as well as several combinations of relaxed negative selection, neutral processes and functional properties throughout the different phases of fixation [3,4]. However, the vast majority of duplicated genes are disabled shortly after the initial duplication events [5], and consequently there is an enrichment of pseudogenes in duplicated regions [6]. Besides the models of evolutionary fate, two special mechanisms, birth-and-death and concerted evolution, also shape the features and organization of duplicated genes [7]. The birth-and-death process caused by recurrent gene duplications and the subsequent disablement of duplicated genes [8] characterizes multigene families and longer time spans, whereas concerted evolution, where the duplicated genes evolve as a unit and in a non-independent way [9], features single pairs of duplicated genes in the same way as multigene families and shorter time spans. In the model of concerted evolution, newly arisen mutations spread through duplicated regions by recurrent non-allelic (ectopic, interlocus, interparalog, interparalogue) gene conversion, which maintains sequence homogeneity among duplicated genes [10].

**Figure 1:**
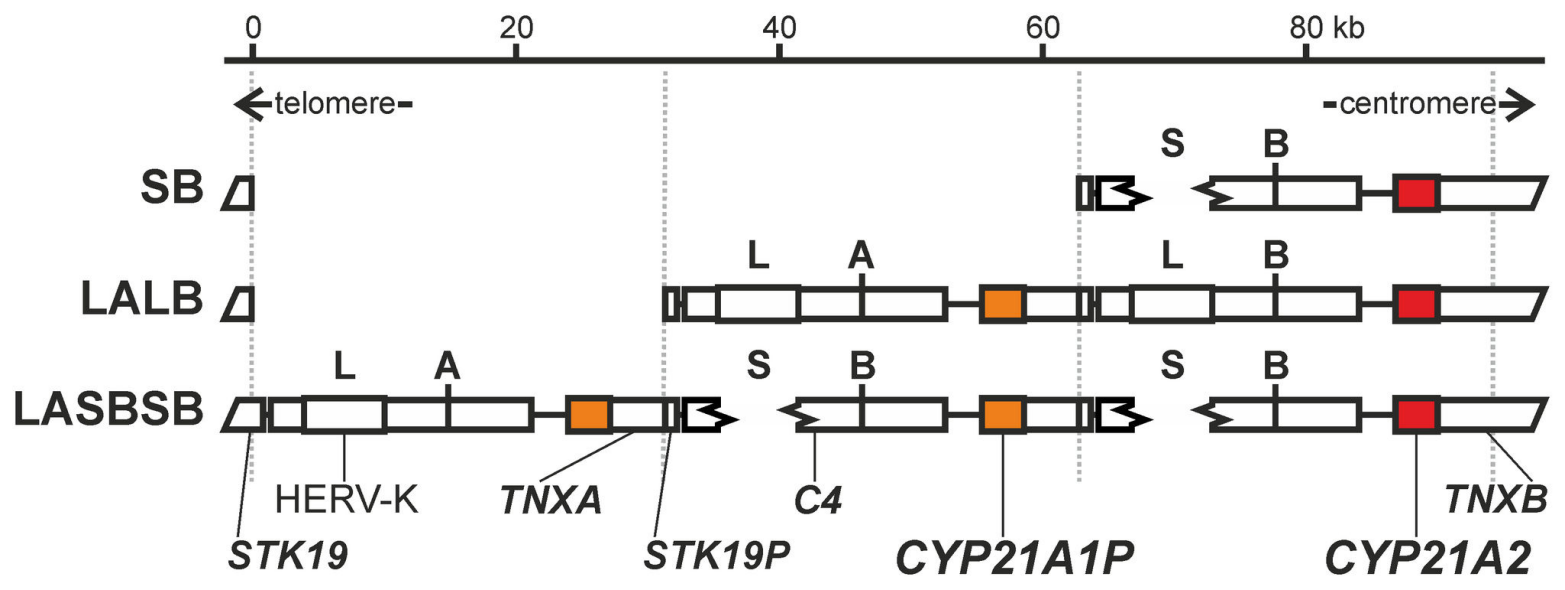
Scaled representation of the organization of human RCCX copy number variation (CNV) depicted by mono-, bi- and trimodular RCCX structure variants. The schematic abbreviations of RCCX structures are indicated on the left side; a module (a repeat) is abbreviated with two letters, the first represents the alleles of HERV-K CNV (L – the long allele or S – short allele [The abbreviation of these alleles comes from the traditional usage of long and short *C4* genes.]), and the second symbolizes the types of *C4* gene (A or B). The duplication of these two letters indicates bimodular RCCX structure, while the triplicate of the two letters means trimodular RCCX structures. Dotted lines indicate the module boundaries, and the directions of the ends of chromosome 6 are indicated by arrows under the scale bar. The variable region of bimodular RCCX contains two pairs of full-length genes, complement component 4 (*C4A* and *C4B*), steroid 21-hydroxylase (*CYP21A1P* and *CYP21A2*), and two pairs of a functional gene and a truncated pseudogene, serine/threonine kinase 19 (*STK19* and *STK19P*) and tenascin-X (*TNXA* and *TNXB*). The illustrated region spans from the telomeric end of exon 4 of *STK19* to the centromeric end of exon 28 of *TNXB*.

The human steroid 21-hydroxylase gene (*CYP21A2*) is responsible for cortisol and aldosterone biosynthesis in the adrenal glands. *CYP21A2* belongs to the *CYP* multigene family, but shows relatively low homology with other members of the family [11]. *CYP21A2* is not located in the large *CYP* gene clusters, but resides a multiallelic, complex and tandem copy number variation (CNV) of the major histocompatibility complex region [12], called RCCX CNV [13,14]. The multiallelic CNVs consist of at least one CNV allele harboring duplicated regions [15], and are regarded as relatively new duplications going through the polymorphic phase of fixation in populations [16]. The most frequent CNV allele of RCCX CNV is bimodular (duplicated), but monomodular and trimodular CNV alleles are also prevalent in humans (Figure 1) [17]. A haplotypic bimodular RCCX structure (CNV allele) encompasses two duplicated pairs of complete genes, the *CYP21* genes, and the complement component 4 (*C4*) genes. In addition to these, the RCCX CNV has a quite complicated organization, and is discussed elsewhere in detail [13].

A haplotypic RCCX structure usually contains one functional gene in the centromeric, 3’-module and zero, one or two disabled pseudogenes (*CYP21A1P*) in the modules towards the telomeric, 5’-direction (Figure 1). Human *CYP21* paralogues (in general, two homologous genes in a duplicated region, in the case of *CYP21* gene, functional gene and pseudogene(s)) show 97-98% nucleotide identity [18,19], and the partial sequences of the *CYP21* paralogues in great apes are more similar to each other than to the orthologues (homologous genes in the same module of a duplicated region, but in different species), indicating sequence homogenization and concerted evolution [20]. The non-allelic gene conversion in the background of the concerted evolution of *CYP21* paralogues is well studied in humans, because congenital adrenal hyperplasia (CAH), a frequent Mendelian disorder mostly caused by gene conversion from *CYP21A1P* to *CYP21A2*, is a focus of human geneticists [21,22]. However, intense conversion activity has been observed at meiotic (equal) crossover hotspots [23], but RCCX CNV is far away from these hotspots, and a low meiotic (equal) crossover rate characterizes its neighboring genomic region [24,25].

Despite the multitude of population genetics literature concerned with the evolution and selection of non-duplicated genes, there are relatively a few genetic studies based on experimental data dealing with the selection and unique evolution processes in duplicated genes (for example: [26-33]). This paucity applies to the duplicated genes of CNVs to a greater extent (for example: [34-36]). Furthermore, the complex organization of RCCX CNV renders the experimental methods more difficult [13], and therefore a population genetics analysis has never been conducted to reveal the polymorphism pattern of *CYP21A2* and the selection forces acting on it. We assumed beforehand that concerted evolution applied to the entire *CYP21A2*, and the polymorphism pattern in the context of the evolution of *CYP21A2* haplotypes was examined. In addition to the human population dataset of *CYP21A2* haplotypes, a human *CYP21A1P* polymorphism dataset and a great ape *CYP21A2* and *CYP21A1P* sequences dataset were also compiled and analyzed.

## Materials and Methods

### Subjects

Genomic DNA samples were collected from 36 healthy, unrelated Hungarian subjects with European ancestry. The study protocol was approved by the Institutional Ethics Committee of Semmelweis University (local ethics committee) and was executed according to the Declaration of Helsinki principles. The subjects gave written informed consent.

### Determination of RCCX CNV structures and *CYP21A2* haplotypes

The two haplotypic RCCX CNV structures of a DNA sample were determined principally in the same way as described recently [13]. Briefly, a set of allele-specific long-range (ASLR) polymerase chain reactions (PCRs) [13], *C4*-type-specific quantitative PCRs (qPCR) [37], and HERV-K CNV allele-specific qPCR [38] were applied to the dissection of RCCX CNV. In addition to these published methods, a *CYP21*-type-specific qPCR was developed for the direct gene copy number determination of *CYP21A1P* and *CYP21A2*. The forward primers were *CYP21*-specific and the same as the primers applied to the nested-PCR (see below). The reverse primer was not allele-specific, and the Taqman minor groove binder probe (Applied Biosystems) was labeled with fluorescent dye 6-FAM (see primer and probe sequences in Table S1). *RPPH1* labeled with dye VIC was used as an endogenous reference (RNase P reference assay, Applied Biosystems). Multiplex reactions were carried out in an Applied Biosystems 7500 Fast Real-Time PCR machine by TaqMan Fast Universal PCR master mix, and the reaction conditions were almost the same as that in the manufacturer's protocol, except the annealing temperature was 64 °C. To check the reliability of the Taqman based *CYP21* gene copy number assay, the *CYP21* qPCR primers were tested on some samples in a LightCycler 1.0 qPCR machine (Roche) by LightCycler FastStart DNA Master SYBR Green I mix using *B2M* and *HMBS* genes as references (Table S1).

To determine the *CYP21A2* haplotypes, *CYP21*-specific nested PCRs were performed from particular ASLR-PCR products as described [13]. Using these nested PCR products, whole-gene haplotypes and genotypic alleles of full-length *CYP21A2* on the *CYP21A2* locus were sequenced mostly as described [13], but with some modified sequencing primers. The 5’-forward and the 3’-reverse sequencing primers (SEQ_11F and SEQ_21R) were moved to the ends of nested PCR products to extend the double-sequenced region to 3357 bp (GenBank NT_007592.15: 31946070-31949426). Two primers (SEQ_15F and SEQ_23F) which lie on some polymorphic sites of *CYP21A1P* were changed to ones avoiding the polymorphisms in *CYP21A1P*, and, finally, the SEQ_12F primer was changed to one which could cover the 5’-end of intron 2 in *CYP21* genes (Table S1). The sequence calls of capillary sequencing were assembled with CLC DNA Workbench v6.5 (CLC bio) and were inspected manually by two different operators.

Because the haplotypic RCCX structures and *CYP21A2* haplotypes could not be experimentally determined from all diploid combination of RCCX structures, bioinformatic haplotype reconstruction by PHASE software v2.1.1 [39,40] was applied to resolve the experimentally indeterminable haplotypic RCCX structures and *CYP21A2* haplotypes from genotypic data [13]. *CYP21A2* haplotype sequences encompassed the whole gene, 122 bp of 5’-flanking region (FR) and 7 bp of 3’-FR. To summarize, a human population dataset of RCCX structure-*CYP21A2* haplotypes was generated from 36 unrelated European subjects by a combined molecular and inferred haplotyping approach.

### Sequence and polymorphism data of *CYP21* genes from great apes

The sequences of human *CYP21A2* haplotype variants were used from a recent study [13], and full-length *CYP21* sequences and intergenic sequences between *C4* and *CYP21* genes (100 bp from the 3’-end of *C4* genes and 400 bp from the 5’-end of *CYP21* gene to avoid the described and potential gene regulatory sites) from human leukocyte antigen (HLA) homozygous cell lines [41] (Table S2). Primate *CYP21* sequences were collected from GenBank, and the status of gene (functional gene or pseudogene) was assessed (File S1). Human *CYP21A1P* polymorphism data was obtained from a previous study (The studied population was German [42], but Hungarians barely deviate from the German population, the European reference population (CEU) or the majority of European populations based on genome-wide polymorphisms [43,44].), but the segregating sites below 0.01 of minor allele frequency were excluded to preclude the possibility of bias, as suggested recently [45]. The polymorphism dataset from the most 3’-end of *CYP21A1P* (NT_007592.15: 31916635-31916691) is lacking, hence the *CYP21A1P* sequences from HLA-homozygous cell lines and a partial *CYP21A1P* sequence (GenBank: KC493621) were checked, but segregating sites were not found in this short segment. To summarize, a great ape *CYP21* sequence dataset and a human *CYP21A1P* polymorphism dataset were compiled.

### Sequence and population data analyses

All alignments were assembled by ClustalX2 v2.0.5 [46], and were edited by CLC DNA Workbench v6.5 and MEGA v5.05 [47]. The Hardy-Weinberg equilibrium and Slatkin's linearized fixation index were calculated by Arlequin v3.5 [48]. Pearson’s chi-square (χ^2^) and continuous Kolmogorov-Smirnov (KS) tests were calculated under the assumption of independent sites and events by STATISTICA 8 (Statsoft). Power for the χ^2^ test was performed with G*Power v3.1.3 [49]. The MEGA v5.05 program [47] was used to construct neighbor-joining (NJ) trees with complete gap deletion and maximum likelihood (ML) trees with gap sites under the assumption that the substitutions followed the Jukes-Cantor (JC) model and uniform rates among sites. The bootstrap tests of NJ and ML trees were performed with 1000 bootstrap replications. Spatial heterogeneity in the phylogenetic signal was detected with the BootScan algorithm built in SimPlot v3.5.1 [50], using a 300 bp window size, 30 bp step size, NJ tree based on the JC model and 1000 bootstrap replications. The Robinson–Foulds tree distance metric [51] was calculated by TOPD/FMTS v3.3 software [52] in order to characterize the similarity among the tree topologies of great ape paralogues and orthologues (The metric skips branch length and bootstrap value, and it evaluates only topology.). Only one human paralogous sequence pair (DBB sequences) was taken into account, because the ambiguous relationships among human *CYP21* sequences were confounding factors for this analysis (It should be noted that the genealogical network is suitable for the analysis of intraspecific sequences with high nucleotide identity [53].). 3Seq [54], BootScan [55], GENECONV [56], MaxChi [57] and RDP [58] algorithms implemented in RDP v3.44 software [59] were chosen to detect potential gene conversion events. The sequences of human *CYP21* genes having high identities were removed following the instructions of RDP v3.44, and a human CYP21 sequence dataset (h08, h21, h30, h35, h41, h42, h44 (QBL), h47, h57, h58, *CYP21A1P* DBB and *CYP21A1P* PGF above a nucleotide difference threshold of 7) and a great ape *CYP21* sequence dataset (h13 (MCF), h28 (COX), h37 (DBB), h44 (QBL), *CYP21A1P* DBB, *CYP21A1P* PGF and all of the chimpanzee, gorilla, orangutan and macaque *CYP21* sequences above a nucleotide difference threshold of 8) were compiled. DnaSP v5.10.01 [60] was applied to perform sliding window analyses using a 300 bp window size and a 30 bp step size. This program was also utilized for assessing the expected haplotype heterozygosity (HHe), the ratio of the nonsynonymous and synonymous average number of pairwise nucleotide differences (π_A_/π_S_), linkage disequilibrium (LD) and neutrality tests; Tajima’s D test [61], Fu’s Fs test [62] and Fay and Wu’s H test [63] were used with the macaque *CYP21A2* sequence as an outgroup if it was needed (H test relies on an outgroup.). Other neutrality tests, normalized Fay and Wu’s H (nH) test [64], Ewens-Watterson (EW) test [65] and the rejection probability of EW test were all carried out using the macaque outgroup by DH software (http://zeng-lab.group.shef.ac.uk). The rejection probabilities of D, Fs, H and nH tests under neutral model and European demography for a 5% significance level were calculated by mlcoalsim v1.42 software [66] based on a previous version of ms software [67]. The rejection probability criteria in DH and mscoalsim softwares were different [65], and the criterion of DH was used. The demography model of European population contained a bottleneck with an effective population size of 1861 from 51 thousand years to 21 thousand years before the present and an expansion from 21 thousand years (about from the retreat of the last glacial age) to the present [68-70]. The gene conversion events were also taken into account omitting the sites of minimum tract length [13] of statistically evident gene conversion events in a similar way to that was described previously [36]. The ratio of the number of nonsynonymous changes per site to the number of synonymous changes (Ka/Ks) and the McDonald-Kreitman test [71] were performed using the gorilla *CYP21A2* sequences (Table S2) by DnaSP v5.10.01 (Hence there were more functional *CYP21A2* coding sequences in gorillas [72] (File S1).).

## Results

### Reconciling the concerted evolution of *CYP21* genes in great apes

**Figure 2:**
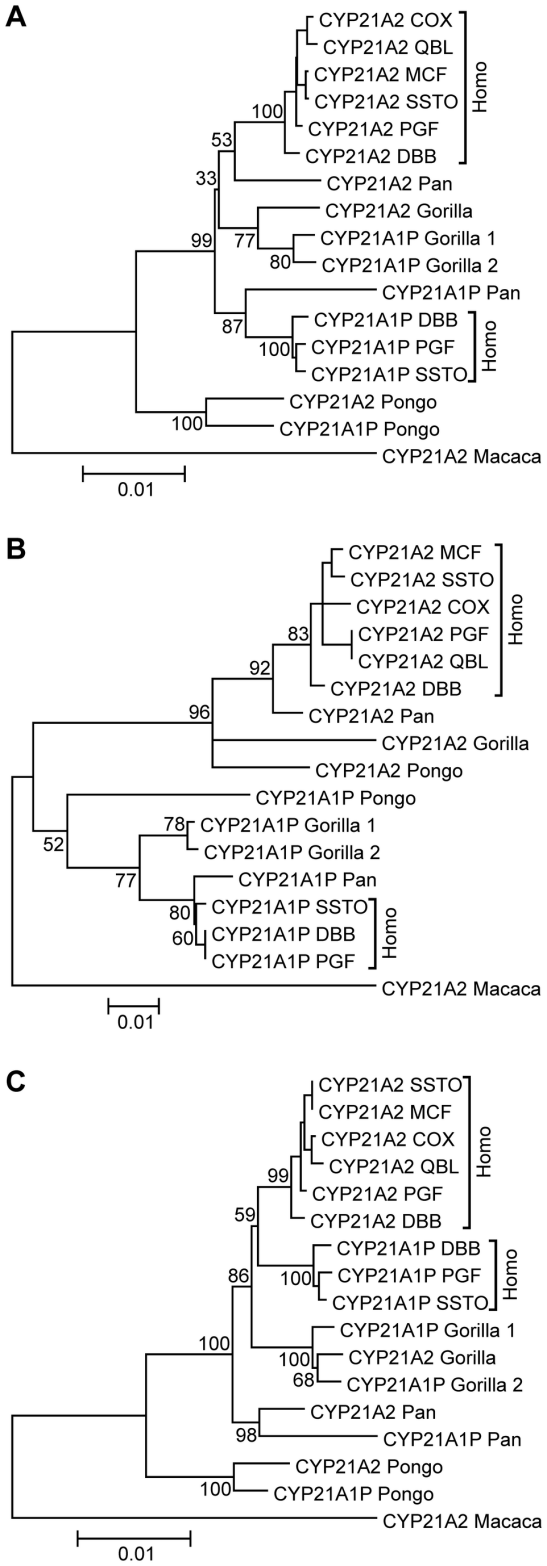
Rooted maximum likelihood phylogenetic trees constructed from selected full-length sequences, intron 2 sequences and *CYP21* gene sequences without intron 2 of the great ape *CYP21* sequence dataset. The names of *CYP21* sequences of HLA-homozygous cell lines available from public databases are represented at the human sequences. Bootstrap values are shown next to the corresponding nodes, but the values within human clades are not presented for clarity. The scale bar indicates genetic distance. (A) Rooted ML tree of great ape *CYP21* full-length gene. (B) Rooted ML tree of great ape *CYP21* intron 2. (C) Rooted ML tree of great ape *CYP21* genes without intron 2.

The concerted evolution of orangutan *CYP21* paralogues has been verified by a phylogenetic tree (paralogues resemble each other better than their orthologues) based on partial sequence data [20], hence this phenomenon was tested on the full-length sequences of the great ape *CYP21* sequence dataset. Agreeing with this, the concerted evolution of orangutan paralogues was unequivocal in both the NJ tree and the ML tree, but the *CYP21A2* sequences of human and chimpanzee were separated from *CYP21A1P* sequences with high bootstrap values (Figure 2A, Figure S1 and S2). Gorilla paralogues resided in their own clade with a moderate bootstrap value, also indicating concerted evolution, and the intercalating position of the gorilla clade could be explained by the incomplete lineage sorting of human, chimpanzee and gorilla [73]. The average nucleotide identities of *CYP21* orthologues and paralogues reflected the relationships of phylogenetic trees (Table S3). For instance, the average nucleotide identity of human and chimpanzee *CYP21A2* sequences was slightly higher than that in human paralogous *CYP21A2* and *CYP21A1P* sequences (98.42% against 97.71%), and, vice versa, *CYP21A1P* orthologues in humans and chimpanzee were slightly closer to each other than chimpanzee *CYP21* paralogues (97.85% against 97.09%).

**Figure 3:**
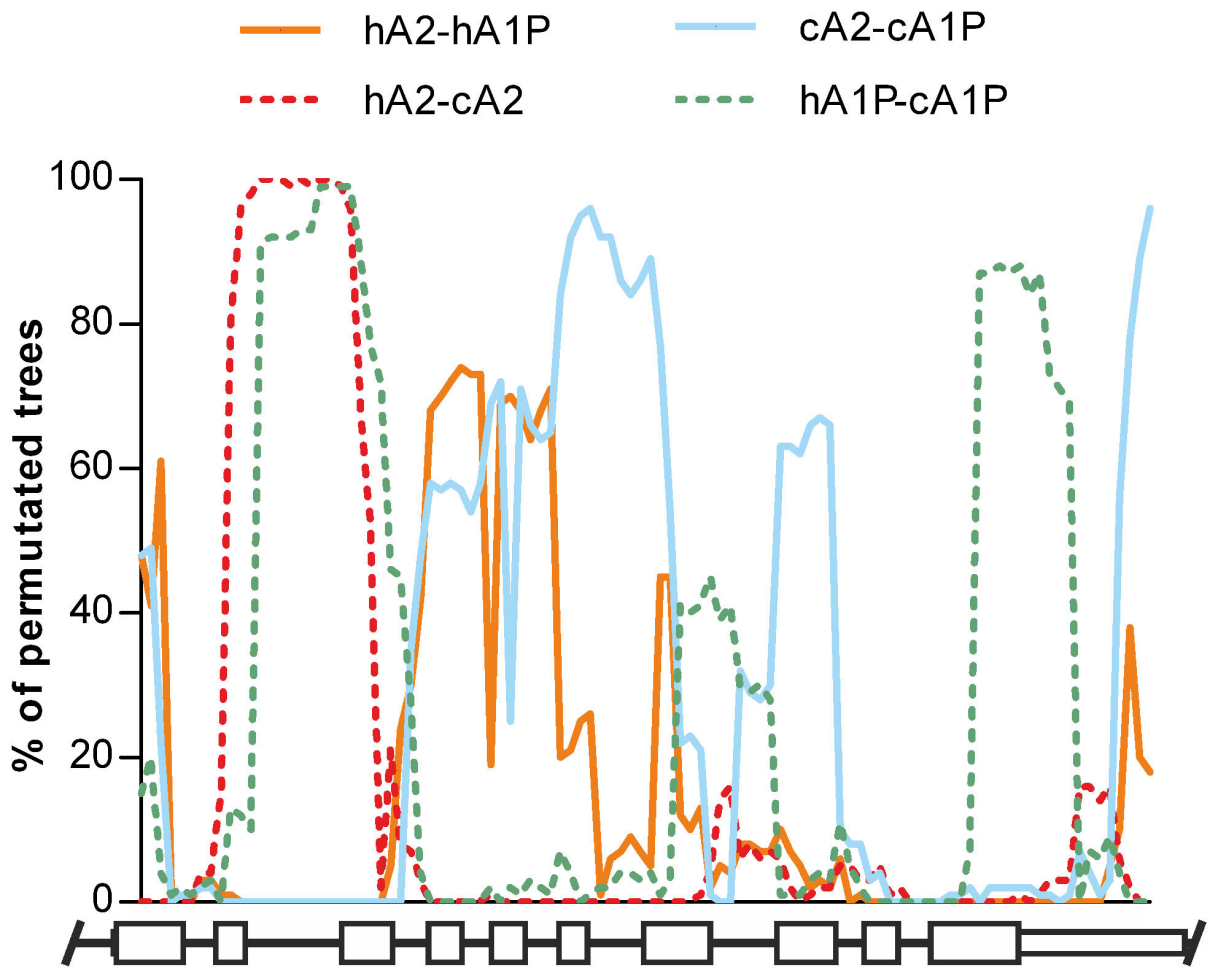
Spatial distributions of phylogenetic signals derived from the different orthologous and paralogous pairs of the human and chimpanzee full-length *CYP21* sequences. Blue line indicates the phlyogenetic signal of human paralogues (hA2-hA1P), red dashed line indicates *CYP21A2* orthologues (hA2-cA2), orange line indicates chimpanzee paralogues (cA2-cA1P) and green dashed line indicates *CYP21A1P* orthologues (hA1P-cA1P). The likelihood of closely related sequences to resemble each other more than random sequences of the same phylogenetic tree is expressed by ‘% of permutated trees’ in y axis. Schematic *CYP21* genes are indicated below the plot, high white boxes symbolize the exons, low white boxes represent the untranslated regions, and black lines indicate the introns and flanking regions.

Because concerted evolution is based on homogenization by gene conversion, the unbalanced sequence transfers between the sections of *CYP21* paralogues could lead to subregions with different evolutionary histories. The great ape alignment was scanned by a BootScan algorithm to dissect whether the spatial heterogeneity of the phylogenetic signal (phylogenetic signal is the likelihood of closely related sequences to resemble each other more than random sequences of the same phylogenetic tree) stayed in the background of straggly phylogenetic relationships between *CYP21* sequences. Surprisingly, the most equivocal phylogenetic signal of human *CYP21A2* was located in a narrow region around intron 2 and was related to the human and chimpanzee *CYP21A2* orthologues indicating that there was no homogenization in this region, whereas the expected signs of homogenization between human paralogues were weaker, and the peaks of the signal were dispersed (Figure 3). The similarity of this phylogenetic signal pattern was demonstrated between the *CYP21A1P* orthologues of human and chimpanzee, and between chimpanzee paralogues; the *CYP21A1P* orthologous signal was very high around intron 2, but the chimpanzee paralogous signal ceased at this section. The signals of the paralogues showed fragmented lines with high peaks in each great ape species (Figure 3, Figure S3). These paralogous signals had different spatial patterns as compared to each other implying the different histories of transitions, but the loss of signal in paralogues around intron 2 proved to be a common feature, and therefore the homogenization was absent from all intron 2 of the great ape *CYP21* genes.

The full-length *CYP21* sequences were divided into two regions based on the spatial distribution of phylogenetic signals; one covered intron 2 and the other encompassed the remaining gene parts. The CYP21A2 and *CYP21A1P* clades were completely separated in the ML tree of intron 2 sequences, showing no signature of concerted evolution or homogenization between paralogous sequences, and the tree followed the model of divergent evolution (Figure 2B). The species in the *CYP21A1P* clade evolved according to the accepted lineage sorting of great apes [73], and only the positions of gorilla and orangutan sequences were vaguely resolved in the *CYP21A2* clade. In the ML tree of the sequences without intron 2, the paralogues from each species were located in separated clades, confirming concerted evolution in each of them (Figure 2C). In addition, the tree topologies of *CYP21* paralogues and orthologues were compared by the Robinson-Foulds metric in ML trees of the full-length gene, the intron 2 subregion and the gene without intron 2. The scale of the metric ranges from 0 to 1 (from complete concordance to complete dissimilarity). The calculated Robinson-Foulds metric was 0.57 for both whole genes vs intron 2 and the whole gene vs gene without intron 2 trees, indicating the medium similarity, whereas the metric was 1 between the trees of the intron 2 and the gene without intron 2, demonstrating the complete lack of similarity (Robinson–Foulds metric range was 0.94-0.95 with 95% conﬁdence interval (CI) of 0.08-0.09 for 100 randomized trees).

To verify the validity of concerted evolution for the coding (cds) region and the non-coding region (non-cds) without intron 2, we further divided the sequences without intron 2 into cds and non-cds subregions, and phylogenetic analyses were performed on them. The clades of different great ape paralogues were separated from each other in both ML trees, however, some discrepancy appeared in the lineage sorting of the gorilla clade (Figure S4). The Robinson-Foulds metric was 0.86 between the cds subregion and the intron 2 subregion, demonstrating very low similarity, while the metric was 1 between the non-cds without intron 2 subregion and intron 2 subregion suggesting complete dissimilartity (for 100 random trees: 0.96 CI: 0.07-0.09). Furthermore, the ML tree of the intergenic region between *C4* and *CYP21* genes also showed the perfect sign of concerted evolution (Figure S4) supporting the idea that the homogenization occurred outside the genes of RCCX CNV as well as inside the *CYP21* genes. The Robinson-Foulds metric was 0.29 for the cds vs. non-cds, the cds vs intergenic and non-cds vs ingergenic trees (for 100 random trees: 0.93-0.96 CI: 0.08-0.09) indicating high concordance. The lack of complete concordance presumably came from the different positions of the gorilla clade and the positions of gorilla paralogues relative to each other.

### Search for statistical evidence of the gene conversion in the *CYP21* sequence

We attempted to reveal the potential gene conversion events using several algorithms implemented in the RDP v3.44 software. Two non-allelic conversions confirmed by more algorithms were detected below the 0.05 significance level in the human *CYP21* sequence dataset for RDP v3.44, but their exact breakpoints were undetermined. One (from *CYP21A1P* DBB to h42, GENECONV p=0.0453, confirmed by 3Seq p=0.0271) was nearly identical with an event of the 3’-untranslated region (UTR) covering the alleles of six adjacent segregating sites on the converted sequence (site 3080, 3102-3186) described recently [13]. The other (from *CYP21A1P* to h41, BootScan, p=0.0182, confirmed by GENECONV p=0.0022, 3Seq p=0.0047) was also very similar to a described event of intron 2 spanning four adjacent sites (site 624-634). In the great ape *CYP21* sequence dataset for RDP v3.44, there was no detected gene conversion from chimpanzee, gorilla, orangutan and macaque *CYP21* sequences to human *CYP21* sequences, and there was an nearly identical non-allelic conversion event of 3’-UTR (site 3080, 3102-3186) without clear breakpoints (from *CYP21A1P* PGF to h37, BootScan p=0.0206, confirmed by 3Seq p=0.0037). The parallel observations of these highly similar gene conversion events in the 3’-UTR originated in one gene conversion event, according to the *CYP21A2* genealogical network [13].

### Spatial distribution of *CYP21* polymorphisms and related indexes

A total of 33 different *CYP21A2* haplotype variants were observed in the human population dataset, including the determination of a new *CYP21A2* haplotype variant (h62, GenBank: KC493622; Table S4). These 33 haplotype variants encoded 6 different protein variants, which consisted of amino acid changes caused by the alleles of 4 segregating sites. If the deletion mutation (rs61338903) affecting three adjacent nucleotides and causing the loss of one amino acid in exon 1 was considered (The deletion mutations in the human population dataset of *CYP21A2* haplotypes were not examined, because they would violate the majority of population genetic models that form the background of the population genetic analyses.), the number of protein variants would not change (Table S5). The validity of experimental data on haplotypic RCCX CNV structures and *CYP21A2* haplotypes was checked, and only the segregating sites of the human population dataset containing a total of 64 *CYP21A2* haplotypes could be consider as segregating sites, because other sites and site frequencies, which have been disclosed in a recent study [13], did not derive from a homogeneous population (File S1, Table S6).

**Figure 4:**
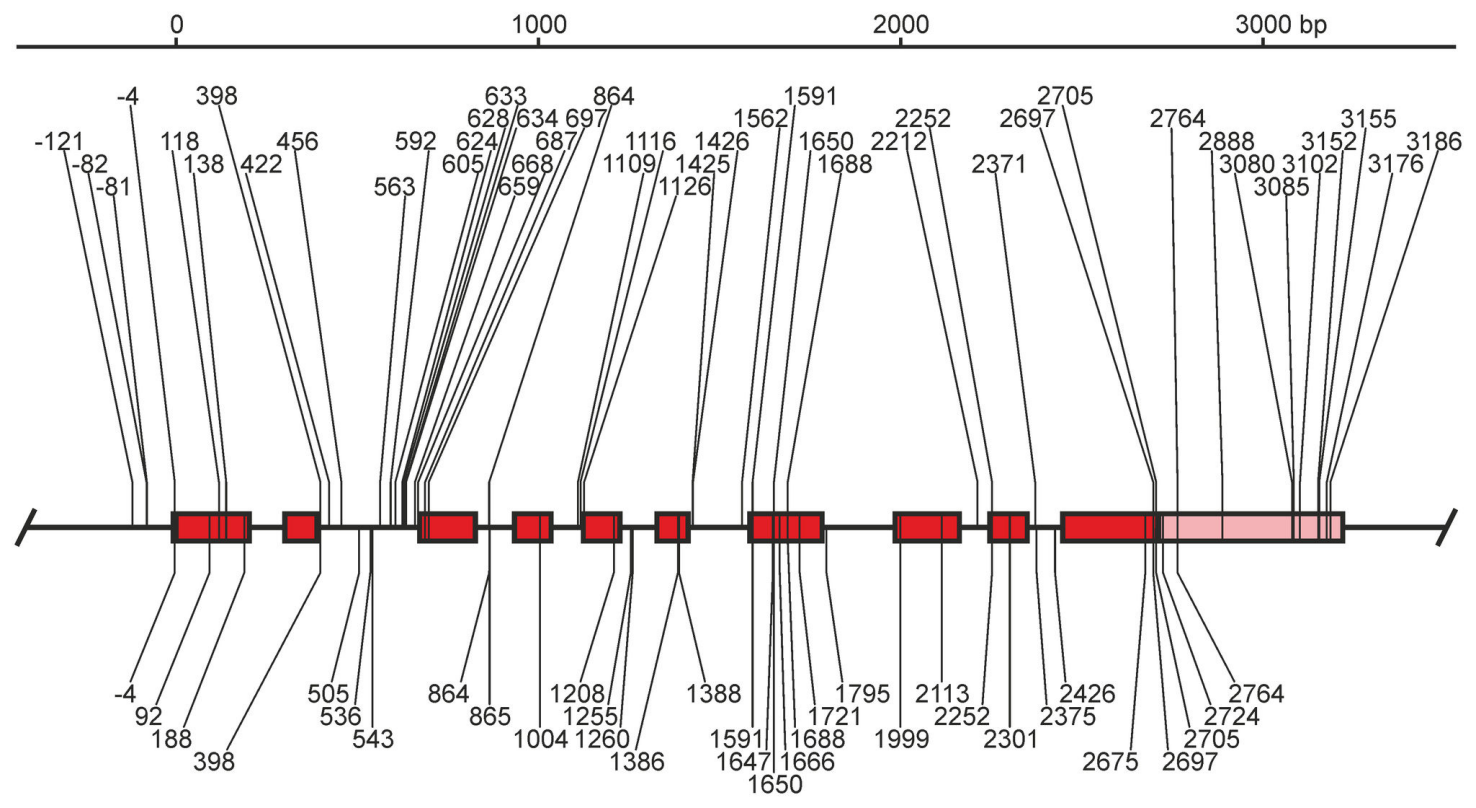
Scale representation of the segregating sites of human *CYP21* genes. Boxes symbolize the exons, red indicates the coding region, pink shows the untranslated regions. Segregating sites are denoted by their position, numbered from the start of the *CYP21A2* coding region in the sequence NT_007592.15: 31945792-31949720. The segregating site of the *CYP21A2* gene can be seen above the depicted gene. The segregating site of the *CYP21A1P* gene derived from an external dataset can be found below the depicted gene.

**Figure 5:**
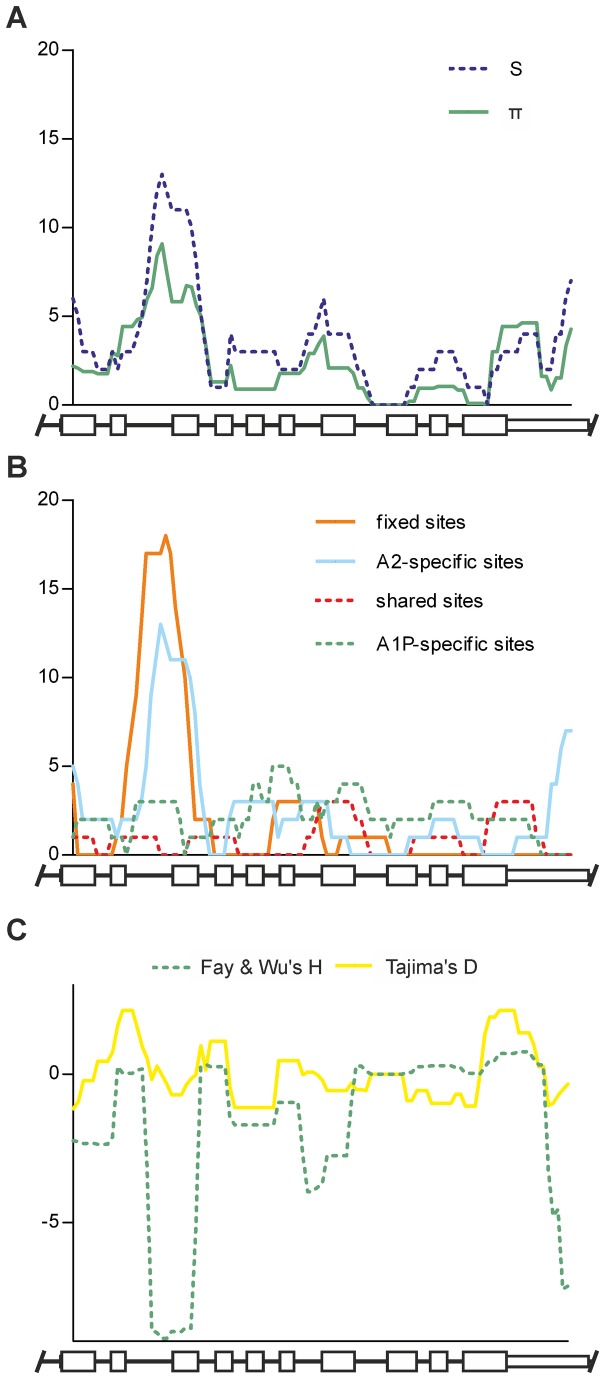
Sliding window plots of different genetic features. Schematic *CYP21* genes are indicated below the plots, high white boxes symbolize the exons, low white boxes represent the untranslated regions, and black lines indicate the introns and flanking regions. (A) Spatial distributions of the number of polymorphic sites (S) and the average number of pairwise nucleotide differences (π) calculated from the population dataset of human *CYP21A2* haplotypes. (B) Spatial distributions of polymorphic site classes (fixed, shared, *CYP21A2*-specific, *CYP21A1P*-specific) based on their coexistence in the human paralogues and calculated from the datasets of human *CYP21A2* haplotypes and human *CYP21A1P* polymorphisms. (C) Spatial distribution of Tajima’s D and Fay and Wu’s H values calculated from the human population dataset of *CYP21A2* haplotypes.

The association of phylogeny with the different regions might reflect the uneven distribution of polymorphic sites observed along the gene (Figure 4). Hence we further investigated the spatial distribution of the polymorphic sites and related indexes using sliding window analysis. The distribution curves of the number of polymorphic sites (S) and the average number of pairwise nucleotide differences (π) calculated from the human population dataset of *CYP21A2* haplotypes ran parallel to each other (Figure 5A). The curves fluctuated, had a jutting peak around intron 2, and the polymorphic sites were not distributed uniformly along the *CYP21A2* gene (KS: p<0.01, χ^2^: p<0.0001). For the assessment of duplicated genes, the classification of polymorphic sites based on their coexistence in paralogues is a routine procedure [74]. Therefore, the sites from the datasets of human *CYP21A2* haplotypes and human *CYP21A1P* polymorphisms were classified into four types: *CYP21A2*-specific and *CYP21A1P*-specific polymorphic sites, at which polymorphisms were observed in either of the two genes; shared sites, at which polymorphisms were shared by the two paralogues; and fixed sites, at which each paralogue had different fixed alleles (Table S7) [75]. The curves of *CYP21A2*-specific and fixed sites had a peak around intron 2 that was much larger than the fluctuating baseline, while the curves of *CYP21A1P*-specific and shared sites did not have any sharp peaks (Figure 5B). Agreeing with this, the distribution of *CYP21A2*-specific and fixed sites deviated significantly from uniform distribution (both sites: KS: p<0.01, χ^2^: p<0.0001), whereas the distribution of *CYP21A1P*-specific and shared sites did not deviate significantly (KS: non-significant (ns), χ^2^: p=0.2730 and KS: ns, χ^2^: p=0.1009). Some neutrality tests detecting skewness from the neutral distribution of allele frequencies in segregating sites [76] were also calculated from the population dataset of human *CYP21A2* haplotypes, and plotted (Figure 5C). The curve of Tajima’s D fluctuated around the null point along the full gene, but the curve of Fay and Wu’s H dropped sharply around intron 2, indicating that the excess of high-frequency polymorphisms, and not just the excess of *CYP21A2*-specific and fixed sites, characterized this subregion.

### Genetic features of the whole *CYP21A2*, intron 2, coding and non-coding without intron 2 subregions

Taking into account the results of the phylogenetic analyses and the spatial distributions of various genetic characteristics in *CYP21A2*, we further used the three subregions; an intron 2 subregion, a cds subregion and a non-cds without intron 2 subregion, and assessed the population genetic features of both the whole gene and the subregions using the population dataset of human *CYP21A2* haplotypes. From all 44 polymorphic sites, 12 sites were observed in intron 2, 21 in the non-cds without intron 2 subregion and 11 in the cds subregion (Table 1). Haplotype heterozygosity was close to its maximum value in the whole gene (HHe=0.949, standard deviation (sd)=0.014), and its values were also high in the different subregions, but the value in intron 2 was only slightly larger than those in the cds and non-cds subregions. As expected from the spatial distribution of the average number of pairwise nucleotide differences, intron 2 and two other subregions showed different nucleotide diversity levels (π), and intron 2 was about 4 times more diverse than the remaining part of the gene. The occurrences of the fixed, shared and *CYP21A2*-specific sites in the subregions highly deviated from each other. This stunning difference in the numbers of sites of the three site classes were highly significant between intron 2 and the remaining part of the gene (χ^2^: p=0.0055, power=1.0000), and the differences between the subregions were also statistically significant (intron 2 vs. non-cds without intron 2, χ^2^: p=0.0106, power=1.0000, intron 2 vs. cds, χ^2^: p=0.0091, power=1.0000, cds and non-cds without intron 2, χ^2^: p=0.0423, power=0.9989).

**Table 1:** Genetic features of the full-length *CYP21A2* gene and its subregions.

	length	GC content	S	HHe	π (x10^-3^)	fixed sites	shared sites	CYP21A2-specific sites	D	Fs	H	nH	EW
full-length gene	3357	0.62	44	0.949	2.55	27	10	34	-0.271 (0.200)	**-9.20 (0.024**)	-22.18 (0.063)	**-3.83 (0.039**)	**0.103 (0.008)**
intron 2 subregion	282	0.54	12	0.885	8.97	**17**	**1**	**11** ^a,b,c^	-0.019 (0.197)	**-6.43 (<0.001**)	**-8.49 (0.026**)	**-4.73 (0.013**)	0.293 (>0.10)
non-cds with-out intron 2 subregion	1587	0.64	21	0.763	2.06	**5**	**3**	**18^*d*^**	-0.832 (0.127)	-4.40 (0.028)	-8.87 (0.091)	-2.94 (0.072)	**0.249 (0.012)**
cds subregion	1488	0.62	11	0.860	1.87	**5**	**6**	**5**	0.542 (0.407)	**-6.68 (0.009**)	-4.82 (0.086)	-2.72 (0.074)	0.153 (>0.10)

S – the number of segregating sites, HHe – haplotype diversity, π – nucleotide diversity, D – Tajima’s D test, Fs – Fu’s Fs test, H – Fay and Wu’s H test, nH – normalized Fay and Wu’s H test and EW – Ewens-Watterson test. Bold characters indicate significant values, and the rejection probability values are shown in parentheses. The rejection probabilities of D, Fs, H and nH were corrected by the demography model of the European population, and rejection probabilities of the Ewens-Watterson test were under a neutral model. **^*a*^**Pearson’s chi-square (χ^2^) test of fixed, shared and *CYP21A2*-specific sites between intron 2 and the remaining part of the gene, p=0.0055, power (χ^2^)=1.0000. **^*b*^**χ^2^ test between intron 2 and the non-cds subregion without intron 2, p=0.0106, power=1.0000. **^*c*^**χ^2^ test between intron 2 and the cds subregion, p=0.0091, power=1.0000. **^*d*^**χ^2^ test between the non-cds subregion without intron 2 and the cds subregion, p=0.0423, power=0.9989.

In neutrality analyses, the selective neutralities of the whole gene and different subregions were first examined using Tajima’s D test. The D test gave no significant values, but there was deviation between the values of the different subregions (Table 1). Rejection probabilities under both neutral and European population demography models were investigated (Table S8). To consider the gene conversion, an additional dataset of human *CYP21A2* haplotypes was generated by omitting the segregating sites affected by statistically evident gene conversion events, and rejection probabilities were also examined. In contrast to D test, Fu’s Fs test and the normalized H test, based on the rejection probabilities corrected with demography, produced significant evidence for the non-neutral distribution of the observed allele frequencies in the whole gene (Table 1). The EW test was also significant under neutral model of the DH software (the EW test was also significant under Slatkin’s implementation of neutrality [77]). For the subregions, the P-values of the Fs test under demography model could be accepted as significant in all subregions, while P-values of the H and nH tests were highly significant only in the intron 2 subregion. Rejection probability values of the EW test were significant only in the non-cds without intron 2 subregion. The significances of rejection probabilities in the dataset of omitted sites affected by gene conversion were identical to those of the human population dataset of *CYP21A2* haplotypes with the demography model except for the H and nH tests of the intron 2 subregion and the H test of whole gene (Table S8). In addition, the synonymous and nonsynonymous polymorphic sites were also assessed in the cds subregion; π_A_/π_S_ was 0.288, Ka/Ks was 0.389, and the McDonald-Kreitman test did not show any deviation.

## Discussion

There are a relatively few theoretical simulation studies on duplicated genes [16,74,75,78], and their study and analysis methods are still in their infancy. In addition, some analysis methods for non-duplicated genes cannot be used for duplicated genes (for instance, Hudson, Kreitman, and Aguade’s test [75]). Despite the youth of the research field, some studies provide good examples for the utilization of methods for duplicated genes: Phylogenetic trees were commonly utilized for the detection of concerted evolution of paralogues [26,31-33]. Spatial heterogeneity in the phylogenetic signal were used for the identification of regions under different forces in a previous article [28], and it should be noted that the algorithms for phylogenetic signal analysis are suited to the detection of recombination [79]. The analyses of fixed, specific and shared site classes can be achieved in the datasets of more studies [26,27,29,30,75], and these results can therefore be compared with the result of the current study.

A total of 33 *CYP21A2* haplotype variants encoding 6 protein variants were determined from a European population by a combined molecular and inferred haplotyping approach. As the organization of RCCX CNV is highly complex, the complicated methods ensured the collection of reliable genetic data as normal as the data of non-duplicated genes if we set the advantage of molecular haplotyping aside. The *CYP21A2* gene was proven to be highly diverse. The haplotype diversity of *CYP21A2* (HHe=0.949) was higher than those observed in the comprehensive gene resequencing studies in humans (0.012-0.929 in 313 genes [80] and 0.360-0.910 in genes with <10 kb length from 100 genes [81]), and the nucleotide diversity of *CYP21A2* (π=2.55x10^-3^) was also much higher than average in humans (0.58x10^-3^ in 313 genes [80] and 0.67 x10^-3^ in 212 genes [82]). The occurrence of fixed, shared and specific sites resembled that in a previous study on duplicated human rhesus genes [29], and this study as well as a theoretical study [78] has predicted mild non-allelic gene conversion activity to be assigned to this kinds of occurrence. The low level of LD between the segregating sites (File S1) also reflected this recombination activity.

Neutrality tests based on different genetic features presented the significant signature of positive selection of the whole *CYP21A2* gene. The population bottleneck and exponential growth characterizing the demographic history of Europeans [68,69] were built into the rejection probabilities of neutrality tests because neutrality tests are sensitive to these population changes (Tajima’s D and Fu’s Fs test to population growth [62], Ewens-Watterson test to bottleneck [65] and Fay and Wu’s H to recent bottleneck [83]) aside from positive selection. Although Tajima’s D test did not show significance, Fu’s Fs test, which detects the frequency spectrum of sites with rare alleles (as well as Tajima’s D test), was significant agreeing with the result that Fs test is more powerful for positive selection than Tajima’s D [62]. The Ewens-Watterson haplotype test, which is conditional on the number of haplotypes, also rejected the hypothesis of neutrality, although the EW test was not tested against the population changes. The normalized Fay and Wu’s test under the demography model presented the significant rejection of neutrality in whole gene, but the H test failed to do this. Both H values were around the threshold of rejection probability, and the deviation in the extent of significances between the Fs and nH tests might be that because the sensitivity of the H test to positive selection (hitchhiking) does not persist long after the fixation of an advantageous allele [84]. Besides the population changes, gene conversion may also confound the results of the neutrality tests, and two independent non-allelic gene conversion events between *CYP21* sequences were evident by detection algorithms. We attempted to build these into the rejection probabilities of neutrality test, but models with gene conversion are not well developed [36], and some available programs did not fit with the observed features of the *CYP21A2* gene (for example, only interallelic (allelic, intralocus) gene conversion (conversion between orthologous sequences) is incorporated in the ms coalescent simulation tool [67], but it does not handle the non-allelic gene conversion.) Therefore, our (rough-and-ready) approach was similar to another CNV study on positive selection [36], and the sites affected by the two non-allelic gene conversion events exempted. The omitted sites were only influenced the significance of the H test in the whole gene, where its value became significant. The rejection of neutrality by the H but not by the D test is the unique signature of recent positive selection [63]. The significant result of the three neutrality tests is confirmed by the fact that a recent positive selection on human *CYP21A2* has also been observed based on fixation index in a genome-wide CNV study [85].

The effect of recent positive selection (adaptive protein evolutions) on the cds subregion was not detected by the McDonald-Kreitman test, but the values for synonymous and non-synonymous nucleotide diversity and divergence (π_A_/π_S_=0.288 and Ka/Ks=0.389) fell below the average values of genomes (π_A_/π_S_=0.34 [86], Ka/Ks (between human and gorilla)=0.42 [87]). These values reflect the predominant negative selection throughout the human genome [88-90]), and indicated a weak purifying (negative) selection on *CYP21A2*, and this finding was in accord with the significant negative selection in the protein coding sequence of *CYP21A2* described by a previous genome-wide study [88]. The presence of purifying selection is not surprising in the case of *CYP21A2* and CAH, hence the purifying selection called in the formal literature is often genetic disease when the mutation affects humans [91]. The recently published three-dimensional crystal structure of CYP21A2 protein [92] has indicated that amino acid residues maintaining the enzyme structure are distributed throughout the entire structure, and that negative selection may affect the majority of the coding region. Furthermore, the cds subregion and the non-cds without intron 2 subregion also differed in the occurrences of fixed, shared and *CYP21A2*-specific sites, implying that the occurrences of site classes was actuated by the weak negative selection.

In addition to the negative selection on the cds subregion of *CYP21A2*, a line of evidence from independent datasets verified the separation of genetic features of intron 2 from those of the remaining part of the gene. First, the clades in the phylogenetic tree of intron 2, which could be demonstrated only with primate sequences available from public databases, followed divergent evolution, whereas the remaining part of the gene showed the signs of concerted evolution. The latter applied separately to the cds and the non-cds without intron 2 subregions, and moreover, to the intergenic region between *C4* and *CYP21* genes, and the values of the Robinson–Foulds tree distance metric supported the observed similarities and dissimilarities between the tree topologies. It should be noted that there is little functional or population data from the RCCX CNV of great ape monkeys. For example, the functional and disabled states of different gorilla and orangutan paralogues has not been functionally confirmed, but the sequences applied in this study agreed well with the independent sequences from a previous study [20]. Second, the occurrence of fixed, shared and *CYP21A2*-specific sites, which was classified based on only the genotypic data of polymorphic sites of human paralogues, and was free from the bioinformatic haplotype reconstruction, showed significant difference in intron 2 compared to those in the remaining part of the gene and the other two subregions. A clear excess of the fixed sites, which feature the reduction of effective non-allelic conversion rate and homogenization due to selection [74], were observed in intron 2. The presence of the fixed sites and the potential reduction of effective non-allelic conversion in intron 2 were in concordance with the lacks of homogenization and concerted evolution, however, one of the statistically evident gene conversion event occurred in intron 2 subregion. Third, the Fs, the H and normalized H tests, which were calculated from the human population dataset of *CYP21A2* haplotypes, deviated from neutrality in intron 2 under the European demography model. At first glance, the EW test value of intron 2 contradicted the presence of positive evolution, but observed homozygosity was slightly higher in intron 2 despite the fact that the intron 2 haplotype adequately characterized the full-length haplotype, and the expected homozygosity is conditional on the number of sites, which can greatly change the critical value of shorter sequences with fewer sites. Along the same lines, omitting one-third of segregating sites because of the observed gene conversion in the intron 2 subregion could also affect the rejection probabilities of the H tests, and may not necessarily influence the rejection probabilities in a direct way. We conclude that positive selection presumably focused on intron 2, however, the selective sweep of neutral sites partly slurred and spread the signature of positive selection across the whole *CYP21A2* gene. Therefore, the positive selection, which shapes the diversity and divergence of intronic DNA in eukaryotes [93,94], was potentially associated with the excess of fixed and *CYP21A2*-specific sites in intron 2. In accord with this, the accumulation of fixed sites has been connected to the positive selection site of coding sequences in tandem duplicated genes, although both duplicated genes are functional in these cases [28,29].

Besides the detected positive selection in *CYP21A2*, RCCX structures harboring only the *C4A* gene (one of the two types of *C4*) are associated with different hormone levels [95], implying that the *CYP21A2* haplotype variants can function differently, and some variants may be advantageous. The search for the causative site or sites under positive selection should be conducted mostly in intron 2, which raises the question as to what the functional role of the potential site or sites may be. Gene regulation could be a plausible answer to this question, since an alternative transcript retaining intron 1 and 2 is expressed from *CYP21A2* with a relative abundance of 10-20% compared to the correct transcript [96]. Other alternative transcripts have also been recognized [97], and their abundance and frequency suggest that these alternative transcripts are not aimlessly generated and may contribute to alternative splicing. Furthermore, the *CYP21A2*-specific site 659 (rs6467) was located at the same site as one of the most frequent CAH mutations (an third allele of this site) affecting RNA splicing [22]. Therefore, positive selection may drive the recent adaptive change of cortisol and aldosterone responses through the gene regulation of *CYP21A2*. This matches well with the fact that genes having environmentally responsive functions are amassed in CNVs with duplicated genes, and these genes have long been considered to be subject to rapid adaptive evolution [98].

## Supporting Information

Table S1 **Primers used in the study.** Allele-specific sites are indicated on the sequences by underscore. Non-allele-specific primer sequences avoid the polymorphic sites based on the ENSEMBL database and the sequences of six HLA-homozygous cell lines.
(DOC)

Table S2 **Sequences from GenBank used in this study.**
(DOC)

Table S3 **Average nucleotide identities of higher primate (*Catarrhini*) *CYP21* orthologues and paralogues.** Minimum and maximum identity values are shown in parentheses. hA2 indicates human *CYP21A2* sequences, hA1P indicates *CYP21A1P* sequences, c, g, o and m before A2 or A1P indicates chimpanzee, gorilla, orangutan and macaque sequences, respectively.
(DOC)

Table S4 ***CYP21A2* haplotype variants and their segregating sites.** RCCX structures assigned to a particular haplotype are indicated in column 2. Column 3-56 indicate the segregating sites, bold numbers indicate the segregating sites of the current study, normal numbers indicate the sites being absent in the current study, but described in a recent study [13]. The concordance with ancestral MHC haplotype from external database (GenBank) is indicated in column RCCX. Four variants of RCCX structure-*CYP21A2* haplotype variants agreed with the sequences of COX, DBB, MCF and QBL cell lines. The *CYP21A2* sequence in the PGF cell line deviated from the h58 haplotype by one nucleotide (PGF -1).
(XLS)

Table S5 **Protein variants were encoded by *CYP21A2* haplotype variants in the current study.** Protein variants were derived from six segregating sites causing amino acid changes; site 28-30 (rs61338903, amino acid (aa) 12) --- – -, CTG – leucine (L), site 687 – (rs6474, aa 102) A – lysine (K), G – arginine (R), site 1650 (rs6472, aa 286) C – threonine (T), G – serine (S), site 1688 (rs6471, aa 281) G – valine (V), T – leucine (L) and site 2705 (rs6473, aa 493) A – asparagine (N), G – serine (S). The site 28-30 was not included in the genetic analyses of the current study.
(DOC)

Table S6 **Frequencies of haplotypic RCCX structure variants in the current study and in a recent family-based study [17].** A module (a repeat) is abbreviated with two letters, the first represents the alleles of HERV-K CNV (L – the long allele or S – short allele), and the second symbolizes the types of *C4* gene (A or B). The multiplication of these two letters indicates bi- and trimodular structures. The number in parentheses indicates the number of *CYP21A2* on the particular haplotypic RCCX structure, but one *CYP21A2* gene is not shown.
(DOC)

Table S7 **Segregating sites of human *CYP21* genes and differences between the human paralogues.** The sites of the datasets of human *CYP21A2* haplotypes and human *CYP21A1P* polymorphisms were classified based on their coexistence into four types: *CYP21A2*-specific and *CYP21A1P*-specific polymorphic sites, at which polymorphisms were observed in either of the two genes; shared sites, at which polymorphisms were shared by the two paralogues; and fixed sites, at which each paralogue had a different fixed allele.
(DOC)

Table S8 **Rejection probabilities of neutrality tests.** Rejection probabilities of Tajima’s D test, Fu’s Fs test, Fay and Wu’s H test, normalized Fay and Wu’s H (nH) test and Ewens-Watterson (EW) test were under a neutral model, a demography model of the European population or a European demography model on the dataset without sites affected by gene conversion. Rejection probabilities of Ewens-Watterson test were not calculated under the European demography model, and the values of neutrality tests are not shown for dataset without sites affected by gene conversion. ***^a^***The cds subregion was not affected by statistically evident gene conversion events.
(DOC)

Figure S1 **Rooted neighbor-joining phylogenetic tree constructed from the full-length sequences of the great ape *CYP21* sequence dataset using complete gap deletion.** The names of *CYP21* sequences of HLA-homozygous cell lines available from public databases are represented at the human sequences. Bootstrap values are shown next to the corresponding nodes, but the values within human clades are not presented for clarity. The scale bar indicates genetic distance.
(TIF)

Figure S2 **Rooted maximum likelihood phylogenetic tree constructed from the full-length sequences of the great ape *CYP21* sequence dataset using gap sites.** The names of *CYP21* sequences of HLA-homozygous cell lines available from public databases are represented at the human sequences. Bootstrap values are shown next to the corresponding nodes, but the values within human clades are not presented for clarity. The scale bar indicates genetic distance.
(TIF)

Figure S3 **Spatial distributions of phylogenetic signals derived from the gorilla and orangutan paralogous pairs.** Gorilla pair is indicated by gA2-gA1P, and orangutan pair is indicated byoA2-oA1P. The likelihood of closely related sequences to resemble each other more than random sequences of the same phylogenetic tree is expressed by ‘% of permutated trees’ in y axis. Schematic full-length *CYP21* genes are indicated below the plots, high white boxes symbolize the exons, low white boxes represent the untranslated regions, and black lines indicate the introns and flanking regions.
(TIF)

Figure S4 **Rooted maximum likelihood (ML) phylogenetic trees constructed from selected sequences of the cds subregion, the non-cds without intron 2 subregion and the intergenic region between *C4* and *CYP21* genes.** The names of *CYP21* sequences of HLA-homozygous cell lines available from public databases are represented at the human sequences. Bootstrap values are shown next to the corresponding nodes, but the values within human clades are not presented for clarity. The scale bar indicates genetic distance. (A) Rooted ML tree of the great ape *CYP21* cds subregion. (B) Rooted ML tree of the great ape *CYP21* non-cds without intron 2 subregion. (C) Rooted ML tree of great ape intergenic region between *C4* and *CYP21* genes.
(TIF)

File S1 **Assessment of the status of great ape *CYP21* sequences and the validity of experimental data.**
(DOC)
