# Supplementary material for: Both Positive and Negative Selection Pressures Contribute to the Polymorphism Pattern of the Duplicated Human CYP21A2 Gene
Source: PLoS One. 2013 Nov 29;8(11):e81977. doi: 10.1371/journal.pone.0081977 (PMC3843699; doi:10.1371/journal.pone.0081977)
Supplement: Table S1 — Primers used in the study. Allele-specific sites are indicated on the sequences by underscore. Non-allele-specific primer sequences avoid the polymorphic sites based on the ENSEMBL database and the sequences of six HLA-homozygous cell lines. (DOC) [file pone.0081977.s001.doc]

| ***CYP21* quantitative PCR primers** | **primer sequence** | **length** | **position on NT_007592.15 sequence** |
| --- | --- | --- | --- |
| CYP21A2_F | GACCTGTCCTTGGGAGACTACT | 22 | 31946897-31946918 |
| CYP21A1P_F | ACCTGTCGTTGG|TCTCTGCTC | 21 | 31914170-31914190 |
| CYP21_CN_R | CCAGCCTTACCTCACAGAACTC | 22 | 31914299-31914278  31947035-31947014 |
| **Taqman probe** |  |  |  |
| CYP21_CN_probe | TGCTCCACCACTGGCTCCAT | 20 | 31914267-31914248  31947003-31946984 |
| ***B2M* reference gene primers** |  |  | **position on NC_000015.9 sequence** |
| B2M_CN_F | ACAGTGTAACAGGCAAGGGACT | 22 | 45007555-45007576 |
| B2M_CN_R | CGGATGGATGAAACCCAGACA | 21 | 45007713-45007693 |
| ***HMBS* reference gene primers** |  |  | **position on NC_000011.9 sequence** |
| HMBS_CN_F | GGGACTGTGACCTGGGGACTT | 21 | 118958935-118958955 |
| HMBS_CN_R | GGTACCCACGCGAATCACTCT | 21 | 118959006-118958986 |
| ***CYP21* sequencing primers** |  |  | **position on NT_007592.15 sequence** |
| SEQ_11F_v2 | GACGTCCCAAGGCCAATGAGA | 21 | 31913285-31913305  31946019-31946039 |
| SEQ_12F_v2 | CCAGATGTGGTGGTGCTGAACTC | 23 | 31913761-31913783  31946495-31946517 |
| SEQ_15R_v2 | ACTGCGGCAGGAAGCATGAGA | 21 | 31914790-31914770  31947526-31947506 |
| SEQ_23F_v2 | CCCAGGAGTTCTGTGAGGTAAGG | 23 | 31914273-31914295  31947009-31947031 |
| SEQ_21R_v2 | CAGTGTAACAGGCAAGGGACTGAG | 24 | 31916745-31916722  31949480-31949457 |
