# Supplementary material for: Both Positive and Negative Selection Pressures Contribute to the Polymorphism Pattern of the Duplicated Human CYP21A2 Gene
Source: PLoS One. 2013 Nov 29;8(11):e81977. doi: 10.1371/journal.pone.0081977 (PMC3843699; doi:10.1371/journal.pone.0081977)
Supplement: Table S2 — Sequences from GenBank used in this study. (DOC) [file pone.0081977.s002.doc]

| **species** | **sequence ID** | **from** | **to** | **origin** | **CYP21A1P** | **CYP21A2** |
| --- | --- | --- | --- | --- | --- | --- |
| human | NT_113891.2 | 3476721 | 3480077 | COX |  | 1 |
|  | NT_167245.1 | 3256140 | 3258647 | DBB |  |  |
|  |  | 3258919 | 3262274 |  | 1 |  |
|  |  | 3282511 | 3285015 |  |  |  |
|  |  | 3285286 | 3288642 |  |  | 1 |
|  | NT_167247.1 | 3355525 | 3356535 | MCF | partial |  |
|  |  | 3385915 | 3389267 |  |  | 1 |
|  | NT_007592.15 | 31913336 | 31916691 | PGF | 1 |  |
|  |  | 31946070 | 31949426 |  |  | 1 |
|  | NT_167248.1 | 3267124 | 3270480 | QBL |  | 1 |
|  | NT_167249.1 | 3306046 | 3309401 | SSTO | 1 |  |
|  |  | 3338781 | 3342134 |  |  | 1 |
|  | JN034382 |  |  |  |  | 1 |
|  | JN034383 |  |  |  |  | 1 |
|  | JN034384 |  |  |  |  | 1 |
|  | JN034385 |  |  |  |  | 1 |
|  | JN034386 |  |  |  |  | 1 |
|  | JN034387 |  |  |  |  | 1 |
|  | JN034388 |  |  |  |  | 1 |
|  | JN034389 |  |  |  |  | 1 |
|  | JN034390 |  |  |  |  | 1 |
|  | JN034391 |  |  |  |  | 1 |
|  | JN034392 |  |  |  |  | 1 |
|  | JN034393 |  |  |  |  | 1 |
|  | JN034394 |  |  |  |  | 1 |
|  | JN034395 |  |  |  |  | 1 |
|  | JN034396 |  |  |  |  | 1 |
|  | JN034397 |  |  |  |  | 1 |
|  | JN034398 |  |  |  |  | 1 |
|  | JN034399 |  |  |  |  | 1 |
|  | JN034400 |  |  |  |  | 1 |
|  | JN034401 |  |  |  |  | 1 |
|  | JN034402 |  |  |  |  | 1 |
|  | JN034403 |  |  |  |  | 1 |
|  | JN034404 |  |  |  |  | 1 |
|  | JN034405 |  |  |  |  | 1 |
|  | JN034406 |  |  |  |  | 1 |
|  | JN034407 |  |  |  |  | 1 |
|  | JN034408 |  |  |  |  | 1 |
|  | JN034409 |  |  |  |  | 1 |
|  | JN034410 |  |  |  |  | 1 |
|  | JN034411 |  |  |  |  | 1 |
|  | JQ993310 |  |  |  |  | 1 |
|  | JQ993311 |  |  |  |  | 1 |
|  | JQ993312 |  |  |  |  | 1 |
|  | JQ993313 |  |  |  |  | 1 |
|  | JQ993314 |  |  |  |  | 1 |
|  | KC493621 |  |  |  | partial |  |
|  | KC493622 |  |  |  |  | 1 |
| chimpanzee | NC_006473.1 | 32276887 | 32280243 |  | 1 |  |
|  |  | 32303353 | 32306710 |  |  | 1 |
|  | NC_006473.3 | 32274122 | 32276616 |  |  |  |
|  |  | 32300588 | 32303082 |  |  |  |
| gorilla | CU104668.1 | 76801 | 79303 |  |  |  |
|  |  | 79575 | 82932 |  | 1 |  |
|  |  | 103263 | 105761 |  |  |  |
|  |  | 106033 | 109391 |  | 1 |  |
|  |  | 129742 | 132244 |  |  |  |
|  |  | 132516 | 135872 |  |  | 1 |
| orangutan | NC_012597.1 | 32518129 | 32520811 |  |  |  |
|  |  | 32521081 | 32524402 |  | partial |  |
|  |  | 32484953 | 32487450 |  |  |  |
|  |  | 32487720 | 32491072 |  |  | 1 |
| macaque | NC_007861.1 | 31662415 | 31663092 |  | partial |  |
|  |  | 31663364 | 31666725 |  |  | 1 |
