# Supplementary material for: Both Positive and Negative Selection Pressures Contribute to the Polymorphism Pattern of the Duplicated Human CYP21A2 Gene
Source: PLoS One. 2013 Nov 29;8(11):e81977. doi: 10.1371/journal.pone.0081977 (PMC3843699; doi:10.1371/journal.pone.0081977)
Supplement: Table S3 — Average nucleotide identities of higher primate (Catarrhini) CYP21 orthologues and paralogues. Minimum and maximum identity values are shown in parentheses. hA2 indicates human CYP21A2 sequences, hA1P indicates CYP21A1P sequences, c, g, o and m before A2 or A1P indicates chimpanzee, gorilla, orangutan and macaque sequences, respectively. (DOC) [file pone.0081977.s003.doc]

|  | hA2 | hA1P | cA2 | cA1P | gA2 | gA1P | oA2 | oA1P |
| --- | --- | --- | --- | --- | --- | --- | --- | --- |
| hA2 | 99.67  (99.23-99.97) |  |  |  |  |  |  |  |
| hA1P | 97.71  (97.50-98.01) | 99.76  (99.73-99.82) |  |  |  |  |  |  |
| cA2 | 98.42  (98.24-98.60) | 97.36  (97.33-97.39) |  |  |  |  |  |  |
| cA1P | 96.63  (96.47-96.79) | 97.85  (97.83-97.86) | 97.09 |  |  |  |  |  |
| gA2 | 98.28  (98.18-98.51) | 97.43  (97.38-97.47) | 98.18 | 96.50 |  |  |  |  |
| gA1P | 98.00  (97.86-98.15) | 97.99  (97.86-98.13) | 97.81  (97.80-97.83) | 97.11  (97.03-97.18) | 98.76  (98.75-98.78) | 99.55 |  |  |
| oA2 | 96.97  (96.81-97.11) | 96.07  (96.05-96.08) | 96.96 | 95.52 | 96.75 | 96.47  (96.43-96.52) |  |  |
| oA1P | 96.66  (96.52-96.84) | 96.46  (96.43-96.49) | 96.64 | 95.90 | 96.66 | 96.74  (96.70-96.79) | 98.30 |  |
| mA2 | 93.11  (92.97-93.30) | 92.86  (92.81-92.90) | 93.06 | 92.37 | 92.79 | 93.01  (92.95-93.06) | 93.33 | 93.42 |
