# Supplementary material for: Both Positive and Negative Selection Pressures Contribute to the Polymorphism Pattern of the Duplicated Human CYP21A2 Gene
Source: PLoS One. 2013 Nov 29;8(11):e81977. doi: 10.1371/journal.pone.0081977 (PMC3843699; doi:10.1371/journal.pone.0081977)
Supplement: Table S5 — Protein variants were encoded by CYP21A2 haplotype variants in the current study. Protein variants were derived from six segregating sites causing amino acid changes; site 28-30 (rs61338903, amino acid (aa) 12) --- – -, CTG – leucine (L), site 687 – (rs6474, aa 102) A – lysine (K), G – arginine (R), site 1650 (rs6472, aa 286) C – threonine (T), G – serine (S), site 1688 (rs6471, aa 281) G – valine (V), T – leucine (L) and site 2705 (rs6473, aa 493) A – asparagine (N), G – serine (S). The site 28-30 was not included in the genetic analyses of the current study. (DOC) [file pone.0081977.s005.doc]

| **protein variant** | **related *CYP21A2* haplotype variants** | **prevalence** |
| --- | --- | --- |
| -KSVA | h01-05, h11-13, h17 | 0.359 (23) |
| -KSVS | h06 | 0.031 (2) |
| -KTVS | h08 | 0.016 (1) |
| LRSLS | h34 | 0.063 (4) |
| LRSVS | h23, h26-29, h32, h37-38, h40-41, h43-44, h45, h49, h58, h61-62 | 0.453 (29) |
| LRTVS | h46, h48, h54, h56 | 0.078 (5) |
