# Supplementary material for: Both Positive and Negative Selection Pressures Contribute to the Polymorphism Pattern of the Duplicated Human CYP21A2 Gene
Source: PLoS One. 2013 Nov 29;8(11):e81977. doi: 10.1371/journal.pone.0081977 (PMC3843699; doi:10.1371/journal.pone.0081977)
Supplement: Table S6 — Frequencies of haplotypic RCCX structure variants in the current study and in a recent family-based study [17]. A module (a repeat) is abbreviated with two letters, the first represents the alleles of HERV-K CNV (L – the long allele or S – short allele), and the second symbolizes the types of C4 gene (A or B). The multiplication of these two letters indicates bi- and trimodular structures. The number in parentheses indicates the number of CYP21A2 on the particular haplotypic RCCX structure, but one CYP21A2 gene is not shown. (DOC) [file pone.0081977.s006.doc]

| RCCX structure | current study (n=72) | family study (n=184) |
| --- | --- | --- |
| LA | 0.0694 | 0.0652 |
| LA(0) | 0 | 0.0109 |
| LALA | 0.0833 | 0.0761 |
| LALALB | 0.0139 | 0.0054 |
| LALASB | 0.0139 | 0.0109 |
| LALB | 0.4030 | 0.4400 |
| LALBLB | 0 | 0.0054 |
| LASA | 0.0139 | 0 |
| LASALB | 0.0139 | 0.0054 |
| LASB | 0.1670 | 0.2500 |
| LASB(2) | 0.0139 | 0.0109 |
| LASBSB | 0.0556 | 0.0326 |
| LBLB | 0 | 0.0054 |
| LBSASB(2) | 0.0278 | 0.0109 |
| SASB | 0 | 0.0054 |
| SB | 0.1250 | 0.0652 |
