# Supplementary material for: Both Positive and Negative Selection Pressures Contribute to the Polymorphism Pattern of the Duplicated Human CYP21A2 Gene
Source: PLoS One. 2013 Nov 29;8(11):e81977. doi: 10.1371/journal.pone.0081977 (PMC3843699; doi:10.1371/journal.pone.0081977)
Supplement: Table S7 — Segregating sites of human CYP21 genes and differences between the human paralogues. The sites of the datasets of human CYP21A2 haplotypes and human CYP21A1P polymorphisms were classified based on their coexistence into four types: CYP21A2-specific and CYP21A1P-specific polymorphic sites, at which polymorphisms were observed in either of the two genes; shared sites, at which polymorphisms were shared by the two paralogues; and fixed sites, at which each paralogue had a different fixed allele. (DOC) [file pone.0081977.s007.doc]

| **SNP ID (dbSNP)** | **position on CYP21A2 of PGF** | **A2 major allele** | **A2 minor allele 1** | **A2 minor allele 2** | **A1P major allele** | **A1P minor allele** | **site class** |
| --- | --- | --- | --- | --- | --- | --- | --- |
|  | -126 | C |  |  | T |  | Fixed |
| rs183137942 | -121 | C | T |  | C |  | A2 specific |
|  | -113 | G |  |  | A |  | Fixed |
|  | -110 | T |  |  | C |  | Fixed |
|  | -103 | A |  |  | G |  | Fixed |
|  | -82 | C | T |  | C |  | A2 specific |
|  | -81 | A | C |  | A |  | A2 specific |
| rs6470 | -4 | C | T |  | C | T | Shared |
| rs9378251 | 92 | C |  |  | C | T | A1P specific |
| rs6468 | 118 | C | T |  | C |  | A2 specific |
| rs6464 | 138 | C | A |  | C |  | A2 specific |
| rs9378252 | 188 | A |  |  | A | T | A1P specific |
| rs6462 | 398 | C | T |  | T | C | Shared |
| rs6463 | 422 | C | A |  | C |  | A2 specific |
|  | 435 | A |  |  | T |  | Fixed |
|  | 445 | T |  |  | G |  | Fixed |
| rs6449 | 456 | T | C |  | T |  | A2 specific |
|  | 473 | A |  |  | G |  | Fixed |
|  | 481 | A |  |  | G |  | Fixed |
|  | 489 | A |  |  | G |  | Fixed |
| rs188515168 | 505 | A |  |  | A | G | A1P specific |
| rs192217993 | 516 | T |  |  | G |  | Fixed |
|  | 527 | T |  |  | C |  | Fixed |
|  | 533 | A |  |  | T |  | Fixed |
|  | 536 | C |  |  | C | T | A1P specific |
|  | 543 | C |  |  | C | T | A1P specific |
| rs185054992 | 549 | C |  |  | A |  | Fixed |
| rs190742906 | 563 | G | C |  | G |  | A2 specific |
|  | 575 | G |  |  | T |  | Fixed |
|  | 576 | G |  |  | C |  | Fixed |
|  | 577 | T |  |  | A |  | Fixed |
|  | 581 | G |  |  | A |  | Fixed |
|  | 584 | A |  |  | A |  | Fixed |
|  | 585 | A |  |  | G |  | Fixed |
| rs79249676 | 592 | G | A |  | G |  | A2 specific |
|  | 593 | G |  |  | T |  | Fixed |
| rs6450 | 598 | G |  |  | A |  | Fixed |
| rs6451 | 605 | A | C | G | A |  | A2 specific |
| rs59064806 | 624 | G | A |  | G |  | A2 specific |
| rs6453 | 628 | T | G |  | T |  | A2 specific |
| rs35147842 | 633 | G | C |  | G |  | A2 specific |
|  | 634 | G | A |  | G |  | A2 specific |
| rs6467 | 659 | G | A | C | G |  | A2 specific |
| rs147821751 | 668 | G | A |  | G |  | A2 specific |
| rs6474 | 687 | A | G |  | A |  | A2 specific |
| rs6455 | 697 | G | C |  | G |  | A2 specific |
|  | 706 | C |  |  | G |  | Fixed |
|  | 721 | C |  |  | T |  | Fixed |
| rs6466 | 864 | C | T |  | C | T | Shared |
| rs58693631 | 865 | G |  |  | G | A | A1P specific |
| rs6475 | 1004 | T |  |  | T | A | A1P specific |
| rs1040312 | 1109 | A | C |  | A |  | A2 specific |
| rs1040311 | 1116 | C | T |  | C |  | A2 specific |
| rs1040310 | 1126 | G | C |  | G |  | A2 specific |
| rs59184325 | 1208 | G |  |  | G | A | A1P specific |
|  | 1255 | G |  |  | G | A | A1P specific |
| rs12525076 | 1260 | A |  |  | A | G | A1P specific |
|  | 1380 | T |  |  | C |  | Fixed |
|  | 1385 | T |  |  | A |  | Fixed |
|  | 1386 | C |  |  | C | G | A1P specific |
| rs12530380 | 1388 | T |  |  | T | A | A1P specific |
|  | 1394 | T |  |  | A |  | Fixed |
| rs71552100 | 1425 | G | A |  | G |  | A2 specific |
|  | 1426 | T | C |  | T |  | A2 specific |
| rs6465 | 1562 | C | T |  | C |  | A2 specific |
| rs6477 | 1591 | C | G |  | C | G | Shared |
| rs61732108 | 1647 | C |  |  | C | T | A1P specific |
| rs6472 | 1650 | G | C |  | G | C | Shared |
| rs11970671 | 1666 | T |  |  | T | C | A1P specific |
| rs6471 | 1688 | G | T |  | G | T | Shared |
|  | 1721 | G |  |  | G | A | A1P specific |
| rs6442 | 1794 | G |  |  | C |  | Fixed |
|  | 1795 | G |  |  | G | A | A1P specific |
| rs7755898 | 1999 | C |  |  | C | T | A1P specific |
| rs7769409 | 2113 | C |  |  | C | T | A1P specific |
| rs6461 | 2212 | G | A |  | G |  | A2 specific |
| rs6469 | 2252 | C | T |  | C | T | Shared |
|  | 2301 | G |  |  | G | A | A1P specific |
| rs182037914 | 2371 | T | C |  | T |  | A2 specific |
| rs2242571 | 2375 | G |  |  | G | A | A1P specific |
|  | 2426 | C |  |  | C | T | A1P specific |
|  | 2675 | G |  |  | G | C | A1P specific |
| rs6446 | 2697 | G | A |  | G | A | Shared |
| rs6473 | 2705 | G | A |  | G | A | Shared |
|  | 2724 | C |  |  | C | T | A1P specific |
| rs1058152 | 2764 | C | T |  | C | T | Shared |
| rs1058148 | 2888 | C | T |  | C |  | A2 specific |
| rs415620 | 3080 | C | T |  | C |  | A2 specific |
| rs451652 | 3085 | G | A |  | G |  | A2 specific |
| rs7774739 | 3102 | G | A |  | G |  | A2 specific |
| rs6457475 | 3152 | T | C |  | T |  | A2 specific |
| rs6457476 | 3155 | C | T |  | C |  | A2 specific |
| rs7756934 | 3176 | C | T |  | C |  | A2 specific |
| rs7383707 | 3186 | T | C |  | T |  | A2 specific |
