# Supplementary material for: Both Positive and Negative Selection Pressures Contribute to the Polymorphism Pattern of the Duplicated Human CYP21A2 Gene
Source: PLoS One. 2013 Nov 29;8(11):e81977. doi: 10.1371/journal.pone.0081977 (PMC3843699; doi:10.1371/journal.pone.0081977)
Supplement: Table S8 — Rejection probabilities of neutrality tests. Rejection probabilities of Tajima’s D test, Fu’s Fs test, Fay and Wu’s H test, normalized Fay and Wu’s H (nH) test and Ewens-Watterson (EW) test were under a neutral model, a demography model of the European population or a European demography model on the dataset without sites affected by gene conversion. Rejection probabilities of Ewens-Watterson test were not calculated under the European demography model, and the values of neutrality tests are not shown for dataset without sites affected by gene conversion. aThe cds subregion was not affected by statistically evident gene conversion events. (DOC) [file pone.0081977.s008.doc]

|  | D | | | Fs | | | H | | | nH | | | EW | |
| --- | --- | --- | --- | --- | --- | --- | --- | --- | --- | --- | --- | --- | --- | --- |
|  | neutral | with demography | without sites affected by gene conversion | neutral | with demography | without sites affected by gene conversion | neutral | with demography | without sites affected by gene conversion | neutral | with demography | without sites affected by gene conversion | neutral | without sites affected by gene conversion |
| full-length gene | 0.413 | 0.200 | 0.448 | **0.026** | **0.024** | **0.013** | **0.001** | 0.063 | **0.027** | **<0.001** | **0.039** | **0.018** | **0.008** | **0.021** |
| intron 2 subregion | 0.551 | 0.197 | 0.620 | **0.050** | **<0.001** | **0.003** | **0.001** | **0.026** | 0.198 | **<0.001** | **0.013** | 0.197 | >0.10 | >0.10 |
| non-cds sub-region with-out intron 2 | 0.135 | 0.127 | 0.110 | 0.225 | **0.028** | **0.031** | **0.008** | 0.091 | 0.123 | **0.003** | 0.072 | 0.113 | **0.012** | **0.021** |
| cds subregion | 0.782 | 0.407 | -^a^ | **0.023** | **0.009** | -^a^ | **0.012** | 0.086 | -^a^ | **0.006** | 0.074 | -^a^ | >0.10 | -^a^ |
