# Supplementary figures and images for: Both Positive and Negative Selection Pressures Contribute to the Polymorphism Pattern of the Duplicated Human CYP21A2 Gene
Source: PLoS One. 2013 Nov 29;8(11):e81977. doi: 10.1371/journal.pone.0081977 (PMC3843699; doi:10.1371/journal.pone.0081977)

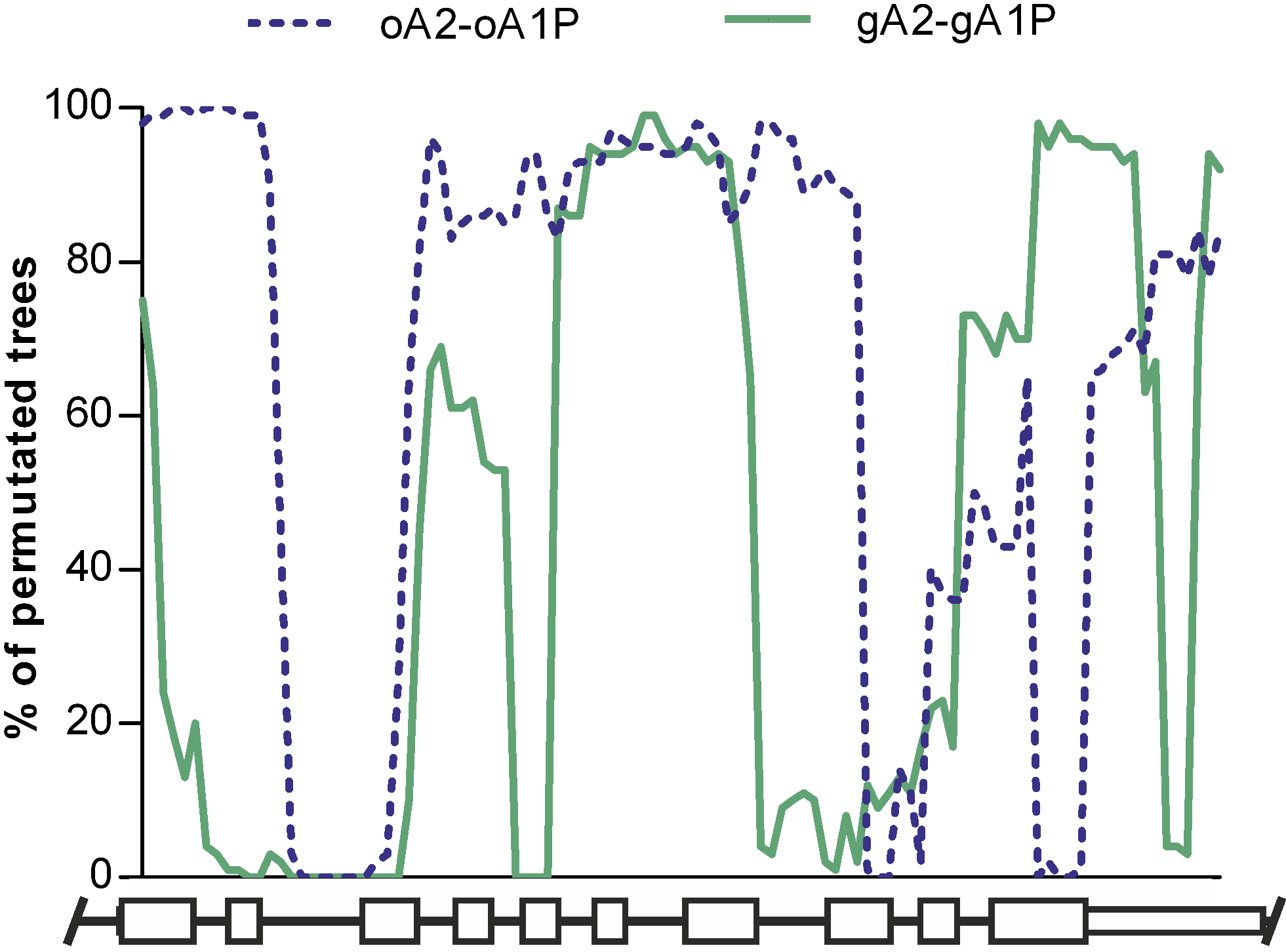

Supplement: Figure S3 — Spatial distributions of phylogenetic signals derived from the gorilla and orangutan paralogous pairs. Gorilla pair is indicated by gA2-gA1P, and orangutan pair is indicated byoA2-oA1P. The likelihood of closely related sequences to resemble each other more than random sequences of the same phylogenetic tree is expressed by ‘% of permutated trees’ in y axis. Schematic full-length CYP21 genes are indicated below the plots, high white boxes symbolize the exons, low white boxes represent the untranslated regions, and black lines indicate the introns and flanking regions. (TIF) [file pone.0081977.s011.tif]
