# Supplementary material for: Both Positive and Negative Selection Pressures Contribute to the Polymorphism Pattern of the Duplicated Human CYP21A2 Gene
Source: PLoS One. 2013 Nov 29;8(11):e81977. doi: 10.1371/journal.pone.0081977 (PMC3843699; doi:10.1371/journal.pone.0081977)
Supplement: File S1 — Assessment of the status of great ape CYP21 sequences and the validity of experimental data. (DOC) [file pone.0081977.s013.doc]

**Assessment of functional and pseudogene status of great ape *CYP21* sequences**

The genomic position and coding region of the *CYP21* sequences in great apes (*Hominidae*) collected from the GenBank database were checked. Chimpanzee (*Pan troglodytes*) *CYP21* paralogues are located in the same position as human paralogues; therefore, the chimpanzee paralogue residing at the telomeric 5’-end of the RCCX CNV is a pseudogene, and the other is the active gene. All gorilla (*Gorilla gorilla*) paralogues have full-length protein sequences (all of them are potentially functional ), hence the paralogue at the centomeric 3’-end of the RCCX CNV (on the human *CYP21A2* locus) was assigned to *CYP21A2,* and the other two were classified as *CYP21A1P* sequences. The orangutan (*Pongo abelii*) paralogue at the centromeric 3’-end of the RCCX CNV has no full-length sequence in databases at present, hence the gap spanning from about the 3’-end of exon 7 to about the 3’-end of exon 9 (NC_012597.1: 32522971-32523515) was completed with an orangutan sequence of the same locus from a previous study . The orangutan paralogue at the telomeric 5’-end of the RCCX CNV encodes full-length protein (on *CYP21A1P* locus), whereas the centromeric 3’-end (chimeric) paralogue was a pseudogene; therefore, the former was assigned to *CYP21A2* and the latter to *CYP21A1P*. A rhesus macaque (*Macaca mulatta*) *CYP21A2* sequence was also used as an outgroup.

**Validity of experimental data on haplotypic RCCX CNV structures and *CYP21A2* haplotypes**

The concordance between the copy numbers of *C4* types, HERV-K CNV alleles and *CYP21* genes was perfect, and the copy numbers agreed with the results of ASLR-PCR and nested PCR in the 36 subjects of this study. New haplotypic RCCX CNV structure was not found as compared with those of a recent family-based study (Table S6). However, two new linkages between RCCX structures and published *CYP21A2* haplotypes occurred. A LALASB RCCX structure (See the denotation of RCCX structure in Figure 1) was assigned to the h13 *CYP21A2* haplotype variant with 0.82 confidence probability, and a LALB structure was assigned to the h08 haplotype variant with 0.67 confidence probability. RCCX structures were in Hardy-Weinberg equilibrium (HWE, p=0.264), and all loci of RCCX structures were also in HWE (p=0.301-1.000). There is no published population dataset of *CYP21A2* haplotypes at this time; therefore, the population data of RCCX structures were compared with the data of a recent study on an independent, healthy Hungarian population . The two populations did not show significant difference (χ^2^: p=0.445; Slatkin's linearized fixation index=0). The *CYP21A2* haplotypes of three subjects (from 36 subjects) who carried RCCX structures with two *CYP21A2* genes were excluded from further analyses, and we did not deal with the RCCX structures in the ‘Results’ section of the study in order to focus on the evolution of *CYP21A2*.

If a *CYP21A2* haplotype was experimentally determined or was resolved by bioinformatic haplotype reconstruction above 0.99 confidence probability threshold, it was regarded as a valid haplotypes. Two *CYP21A2* haplotypes were not valid due to the low confidence probability in 33 subjects with two *CYP21A2* genes on the normal locus of *CYP21A2* (in the 3’-module of RCCX CNV), and these haplotypes were excluded from the further analyses. The sequencing of haplotypes was extended to the 5’-flanking region of the *CYP21A2* gene, and three additional segregating sites (at site -121, site -82 and site -81) were found in this region (Table S4) as compared to the segregating sites of a recent study . The *CYP21A2* haplotypes of this recent study were reexamined, and we found that the minor alleles of two segregating sites were always contained in unique haplotypes (site -121 T in h41, site -81 C in h45). The minor allele of the third segregating site (site -82 T) defined a new haplotype, named h62, which was the same as h29 apart from the T allele of the site -82, and h62 also occurred in the reexamined dataset of a recently published study . Therefore, there was no new haplotype in the current study as compared with those of the recent study, but some haplotypes were lacking due to the smaller sample size. Seven of the missing haplotypes were singletons of the recent study which contained unique alleles (site 426 A in h51, site 505 G and site 516 G in h59, site 523 T in h52, site 549 A in h36, site 865 A in h39 and site 2663 T in h25), whereas two of them contained unique alleles, but harboring haplotypes that occurred more than once (site 598 A and site 2725 A in five h31 haplotypes and site 1794 C in two h14 haplotypes). These lacking segregating sites were not considered as the segragating sites of the population dataset of the current study because those haplotype would not have come from a population dataset (those haplotypes were sorted ). Overall, 64 human *CYP21A2* haplotypes of the population dataset consisted of 44 polymorphic sites, both haplotypes and the separate segragating sites in the population dataset were in HWE (haplotypes p=0.647, sites p=0.226-1.000), and the values of linkage disequilibrium were generally low between the sites (r^2^ average=0.0818 median=0.0096).

**References**

1. Wilming LG, Hart EA, Coggill PC, Horton R, Gilbert JG, et al. (2013) Sequencing and comparative analysis of the gorilla MHC genomic sequence. Database (Oxford) 2013: bat011.

2. Kawaguchi H, O'HUigin C, Klein J (1992) Evolutionary origin of mutations in the primate cytochrome P450c21 gene. Am J Hum Genet 50: 766-780.

3. Banlaki Z, Doleschall M, Rajczy K, Fust G, Szilagyi A (2012) Fine-tuned characterization of RCCX copy number variants and their relationship with extended MHC haplotypes. Genes Immun 13: 530-535.

4. Banlaki Z, Szabo JA, Szilagyi A, Patocs A, Prohaszka Z, et al. (2013) Intraspecific evolution of human RCCX copy number variation traced by haplotypes of the *CYP21A2* gene. Genome Biol Evol 5: 98-112.
